# Chlorhexidine locking device for central line infection prevention in ICU patients: protocol for an open-label pilot and feasibility randomized controlled trial

**DOI:** 10.1186/s40814-020-0564-9

**Published:** 2020-02-18

**Authors:** Nasim Zamir, Makena Pook, Ellen McDonald, Alison E. Fox-Robichaud

**Affiliations:** Thrombosis and Atherosclerosis Research Institute, Hamilton Health Sciences and McMaster University, Hamilton, ON Canada

**Keywords:** Sepsis, CLABSI, Central line infection, Central venous catheter, Device, Prevention, Chlorhexidine, CHG

## Abstract

**Background:**

Critically ill patients in the intensive care unit (ICU) are at risk for central line-associated bloodstream infection (CLABSI) with an incidence up to 6.9 per 1000 catheter days. CLABSI has a significant attributable mortality and increases in-hospital length of stay, readmissions, and costs. Chlorhexidine gluconate (CHG), a broad-spectrum biocide, has been shown to effectively reduce infections including CLABSI; however, few trials have utilized CHG for prevention of central line infections. Our preclinical work has demonstrated a device that diffuses CHG into the intravenous lock solution of central venous catheters and decreases bacterial growth on the catheter lumen. We designed a clinical trial to test the feasibility of using a CHG device in an ICU patient population.

**Methods:**

The proposed pilot trial will be a single centre, open-label, two-arm, parallel group feasibility randomized controlled trial (RCT). Participants will have a central line in situ and will be enrolled within 72 h of admittance to 3 ICUs at a single academic hospital. Exclusion criteria will include suspected infection, chronic indwelling catheters, and CHG allergy. Informed consent will be obtained from eligible participants or their substitute decision maker prior to randomization. Participants will be randomized to receive either usual care or the CHG locking device. Blood cultures will be drawn from all participants every 48 h. The primary objective of this study will be to determine the feasibility of using this protocol to conduct a larger trial. Feasibility will be assessed through the following outcomes: (1) consent rate, (2) recruitment rate, (3) protocol adherence, and (4) comfort level with the device. The secondary objective of this study will be to establish the preliminary efficacy of the device.

**Discussion:**

This study will be the first human RCT to investigate a CHG locking device for the prevention of central line infections. Findings from this trial will inform the feasibility of conducting a large RCT and provide preliminary data on the efficacy of a CHG locking device.

**Trial registration:**

ClinicalTrials.gov, NCT03309137, registered on October 13, 2017.

## Background

### Rationale

Vascular access via central venous catheters (CVCs) is essential for acute patient care in the Intensive Care Unit (ICU) [1]. Despite the utility of CVCs, they have been identified as a risk factor for bloodstream infection that, when not recognized early, can progress to sepsis [2, 3]. CVCs are the most common cause of nosocomial bloodstream infection [4]. A report by the National Nosocomial Infections Surveillance System identified that 87% of all primary bloodstream infections occur in patients with a CVC [5]. Additionally, the relative risk for bloodstream infection is 64 times greater for patients with a CVC compared to patients with only peripheral venous catheters [4]. Instances of bloodstream infection in patients with a CVC are formally referred to as central line-associated bloodstream infection (CLABSI) [1, 6]. Critically ill patients are among those most likely to develop CLABSI [7, 8] with incidence as high as 6.9 per 1000 catheter days [9–12]. Evidence suggests that CLABSI impacts patient mortality and increases in-hospital length-of-stay (LOS) readmissions and costs. A systematic review of 18 studies by Ziegler and colleagues found that CLABSI has a substantial attributable mortality, with an odds ratio of 2.75 for in-hospital death [13]. A 2013 meta-analysis of the burden of hospital-acquired infections (HAIs) in the USA found CLABSI has one of the highest attributable LOS of any HAI at 15.7 days, second only to *Methicillin-resistant Staphylococcus aureus* surgical site infection [14]. A retrospective case-controlled cohort study of 11,802 hospitalizations in the USA reported that 10.4% more CLABSI patients were readmitted to a hospital within 30 days of discharge compared to non-CLABSI patients [15]. CLABSI also presents a significant economic burden, increasing healthcare costs by an average of USD 45,814 per case [14, 16, 17].

Several sources of contamination contribute to the pathogenesis of CLABSI [4]. Pathogens gain access to the external surface of the CVC through transcutaneous migration, and to the luminal surface through contaminated infusate, colonization of the catheter hub, or hematogenous seeding [18]. As interventions to prevent external surface contamination are increasingly used, such as CVC insertion bundles [10, 19], hub colonization has become the dominant route of infection [18]. Once pathogens gain access to a CVC, the lumen acts as a nidus for colonization where bacteria and fungi attach and secrete gelatinous exopolymers, enclosing themselves in a protective matrix called a biofilm [20, 21]. Pathogens growing in a biofilm are particularly resistant to antibiotics and host defenses when compared to free-floating bacteria [20]. Frequent handling of a colonized catheter hub risks displacing the biofilm, potentially leading to bacterial invasion of the catheter inner lumen [22]. This risk may be increased in the ICU where frequent catheter access may occur without adequate hand hygiene or use of alcohol wipes during emergency situations or when staff-to-patient ratios are increased [22].

### Relevant medical literature

Various interventions have been suggested to lower incidence of CLABSI [23], including antimicrobial impregnation of catheters and antibiotic locking solutions. Although impregnated catheters have been shown to reduce CVC colonization, results vary based on setting, and widespread use has not been broadly recommended [24–26]. Antibiotic lock solutions have proven an effective means of decreasing incidence of CLABSI [27]; however, research has shown that their use quickly leads to antibiotic resistance [28]. Recent studies have demonstrated the effective role of topical chlorhexidine gluconate (CHG) for reducing CLABSI [29]. CHG is a broad-spectrum biocide with rapid onset of action, prolonged antimicrobial effects, and excellent efficacy against gram-positive organisms [30]. A prospective, randomized cross-over trial demonstrated that daily bathing with CHG soaked washcloths reduced the incidence of bloodstream infections from 6.60 to 4.78 cases per 1000 patients in the ICU [31]. CHG bathing decreased the likelihood of patients’ resident skin flora entering the bloodstream at the CVC insertion site or extraluminal surface of the catheter, however, manipulation of catheter hubs remains a more prominent source of infection [32]. The antimicrobial efficacy of CHG has also been shown in vitro and in preclinical animal trials in a previous study by our group [33]. CHG significantly reduced *Staphylococcus aureus* contamination, decreasing bacterial load by 6 log_10_ colony-forming units (CFU) in vitro, and by 3–4 log_10_ CFU/lumen in Yorkshire swine with a CVC inserted into the jugular vein [33]. This preclinical work informed the potential of a CHG locking device to decrease incidence of CLABSI and led to the current pilot trial.

### Overall research question

**Fig. 1:**
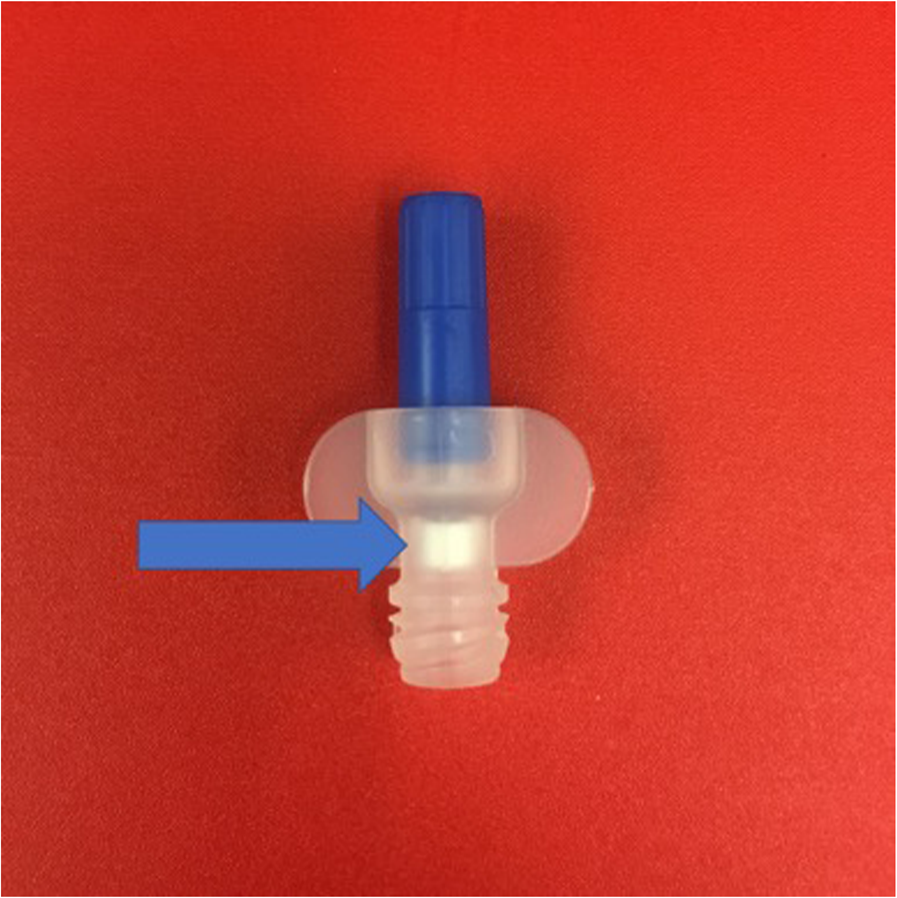
The ChloraLock^TM^ device contains freeze-dried CHG (arrow) that dissolves with introduction of the locking solution

ChloraLock^TM^ (Fig. 1) is a device that instills CHG into the lock solution of venous catheters with the aim of sterilizing solutions passing into the catheter during routine locking procedures. In patients admitted to the ICU with a CVC in situ, does CHG locking solution administered by the ChloraLock^TM^ device reduce risk of intravenous line colonization and improve patient and hospital outcomes by preventing cases of CLABSI?

### Pilot trial research question

Is conducting a large randomized controlled trial (RCT) feasible using this device and protocol?

### Explanation of comparator

There are currently no antimicrobial locking solutions broadly recommended for prevention of CLABSI. Therefore, ChloraLock^TM^ will be compared to ‘usual care’.

## Methods

### Study design

The ChloraLock^TM^ pilot trial is a single centre, open-label, two-arm, parallel-group feasibility RCT. Randomization will be performed with a 1:1 allocation, and study participants and evaluators will not be blinded to intervention assignment due to lack of placebo ChloraLock^TM^ devices. Order of randomization will be concealed by means of random number generator and sealed envelopes (see Allocation and Blinding details below). Patients will be recruited from the ICU at the Hamilton General Hospital (HGH) within 72 h of admission if they have a CVC in situ. The primary objectives of the ChloraLock^TM^ pilot trial are to assess uptake of the trial protocol and ChloraLock^TM^ device by ICU staff and determine the feasibility of conducting a large RCT to investigate our overall research question. Secondary objectives are related to the clinical efficacy of the ChloraLock^TM^ device. ICU nurses will be responsible for using the ChloraLock^TM^ device and operationalizing the protocol; therefore, we will be collecting data on their perceptions of the trial using a voluntary survey. Feasibility and survey data will be used to inform the conduct of a larger RCT.

This protocol adheres to the Standard Protocol Items: Recommendations for International Trials (SPIRIT) guidelines and is formatted in accordance with the SPIRIT 2013 checklist (see Additional file 1) with all required items [34, 35]. The World Health Organization Trial Registration Dataset (see Additional file 2) and a schedule of enrollment, interventions, and assessments (Table 1) are included. Prior to enrolment of our first participant, the protocol was registered at ClinicalTrials.gov (NCT03309137).

**Table 1:** SPIRIT figure detailing enrolment, interventions, and assessment

Timepoint	Study period							
	Enrolment	Allocation	Post-allocation					Close-out
	< 72 h post ICU admission (*-t*_1_)	0	t_1_	t_2_	t_3_	*t*_4_	etc.	intervention discontinued (*t*_*x*_)
Enrolment								
Eligibility screen	X							
Informed consent	X							
Allocation		X						
Interventions								
Usual care		X	X	X	X	X	X	
CHG locking device		X	X	X	X	X	X	
Assessments								
Baseline variables	X	X						
Recruitment rate	X	X						
Consent rate	X	X						
Initiation of study procedures		X						
Protocol adherence			X	X	X	X	X	
Comfort level			X	X	X	X	X	
Central line colonization			X	X	X	X	X	
Bacteremia			X	X	X	X	X	
LOS in ICU								X
LOS in hospital								X
ICU mortality at 28 days								X
Hospital mortality at 28 days								X

### Setting

The ChloraLock^TM^ pilot trial includes three ICUs at the Hamilton Health Sciences General Site, a large academic hospital and major cardiac surgical centre in Hamilton, Ontario, Canada. A single site was selected to determine the feasibility of conducting a larger RCT. In April 2018, the trial was expanded from our cardiac surgical ICU to both medical–surgical ICUs due to slow recruitment rates.

### Participants

Participants are patients admitted to one of our three participating ICUs at the HGH who meet the following eligibility criteria:

#### Inclusion criteria

1. Age greater than 18 years
2. First ICU admission for this hospital stay
3. Patient has ≥ 1 CVC in situ
4. CVC expected to remain in situ ≥ 72 h
5. Less than or equal to 72 hours post-admittance to the ICU

#### Exclusion criteria

1. Expected discharge post-admittance to ICU is ≤ 36 h
2. Hopeless prognosis / limitation of care / palliative measures only
3. Admitted to the ICU with a known or suspected infection and receiving antibiotics
4. Patients with a chronic indwelling central venous catheter
5. Patients with a known CHG allergy

### Interventions

Patients will be randomized in a 1:1 ratio to either (1) usual care, or (2) CHG locking device.

Standard flushing and locking practices of central and peripheral venous catheters will be continued for participants allocated to ‘usual care’. All venous catheters will be flushed with a pre-specified volume of 0.9% Normal Saline (NS) solution ranging from 3 to 30 mL. As per hospital approved protocol, venous catheters will be flushed in a turbulent fashion to promote patency of the internal lumen. Catheters that are not infusing will be capped with a Luer lock adapter and considered locked. An additional movie file shows procedures for participants allocated to usual care in more detail (see Additional file 3).


**Additional file 3** Instructional video on procedures for participants allocated to usual care.


Participants allocated to ‘CHG locking device’ will receive ChloraLock^TM^ instillation addition to usual care. Venous catheters that are not infusing will be flushed with 0.9% NS solution as per hospital-approved protocol. Subsequently, ChloraLock^TM^ will be Luer locked onto a 3-mL 0.9% NS prefilled Posiflush^TM^ syringe, which will be treated as a single-use device to instill a pre-specified volume of CHG locking solution into the lumen of the venous catheter (Table 2). The catheter will then be capped with a Luer lock adapter and considered locked, thereby containing the CHG locking solution within the lumen. For participants in the CHG locking device group, each venous catheter will be labelled with the pre-specified volume of CHG locking solution required to lock the catheter. Before re-accessing the catheter for further sampling or infusions, all fluid will be aspirated to clear the lumen of CHG. An additional movie file shows procedures for participants allocated to CHG locking device in more detail (see Additional file 4).

**Table 2:** Pre-specified flushing and locking volumes

Reference number	Full name	Common name	Flushing volume (mL)	Locking volume (mL)
MED-RX 10-1026	Minibore tubing with slide clamp, thinner lumen extension for peripheral IV	Normal saline lock extension (small bore)	3.0	0.5
MED-RX 10-114RL	Standard bore tubing with slide clamp, thicker lumen extension for peripheral IV	Normal saline lock extension (large bore)	3.0	1.0
Baxter JC 1946	Y-type extension set (1.8cc in total, 1.2cc per line)	Y-normal saline lock extension (per lumen)	3.0	1.0
ARROW SS-14703	ARROW multi-lumen central venous catheter	Triple lumen (per lumen)	10.0	0.5
ARROW SI-09880	ARROW percutaneous sheath introducer set	Cordis (side-arm lumen of introducer)	10.0	2.5
PICC lines	PICC lines	PICC line (per lumen)	20.0	0.5
ARROW CA-22122-F	ARROW temporary two-lumen hemodialysis catheter	Temporary hemodialysis catheter 12Fr (per lumen)	10.0	2.5^a^
MAHURKAR Elite 8888212216	Covidien MAHURKAR elite acute hemodialysis dual lumen catheter kit 13.5Fr	Acute dual lumen hemodialysis catheter 13.5Fr (per lumen)	10.0	2.5^a^

^a^4% sodium citrate solution


**Additional file 4** Instructional video on procedures for participants allocated to CHG locking device.


A single set of blood cultures will be drawn from the CVC of all study participants every 48 h beginning on the day of study enrolment until the patient has all CVCs removed, has a documented infection, or leaves the ICU. Blood cultures will be analyzed for infectious organisms in the core laboratory of the HGH.

The bedside nursing staff will perform the assigned interventions. Bedside nurses will document flushing and instillation of CHG on study-specific logs, which will be checked daily by the research staff. The only exception to the above procedures is flushing and locking of temporary and acute dialysis catheters will be carried out using 4% Sodium Citrate solution rather than 0.9% NS.

We will approach principal investigators (PIs) and research coordinators of all other studies conducted in the ICU to determine co-enrolment eligibility.

### Criteria for discontinuing allocated intervention

For a given trial participant, exit criteria from the study and assigned intervention will be as follow:

1. Consent for ongoing study participation is withdrawn by the study participant or their substitute decision maker (SDM).
2. All venous catheters are discontinued.
3. Participant experiences an adverse reaction to CHG locking solution.
4. Death or discharge from ICU.

### Strategies to improve adherence to intervention protocol

Prior to study initiation regular in-services were held with > 80% attendance to educate the bedside nursing staff on the study protocol and ChloraLock^TM^ device. The vascular access team at the HGH helped develop a flushing and instillation taxonomy for all venous access catheters. Two bedside nurses were identified as ‘Nurse Champions’, their participation in study meetings and leadership qualities ensures they will be well positioned to be liaisons for staff who work weekend and night shifts. Upon study initiation, a one-page handout including detailed instructions for the study intervention will be distributed throughout the ICU. The research staff will visit the bedside of study participants daily to ensure that bedside nurses are educated on the protocol and oversee documentation of protocol adherence. Participants in the CHG locking device group will have all venous catheters labelled with a bright-green tag to indicate the volume of locking solution required. For all study participants, a bright-green sticker will be placed on the front of their medical chart and on the corner of their patient care plan Kardex to alert staff of trial enrolment and assigned intervention. A ‘flushing and locking log’ will be kept at the bedside of all study participants and will require nurses to document each time they access a venous catheter to perform flushing or locking procedures. We will document and report protocol deviations including failure to use ChloraLock^TM^ to administer CHG locking solution, aspirate CHG from a venous catheter upon re-accessing the line, and draw a blood culture from the CVC of a patient every 48 h.

## Outcomes

The primary outcomes of the ChloraLock^TM^ pilot trial relate to the feasibility of conducting a larger RCT.

1. *Consent rate* will be measured as the proportion of patients and SDMs approached who consent to participate in the trial; a successful consent rate will be defined as ≥ 80%. Consent rates will be reviewed on a monthly basis to improve enrolment practices.
2. *Recruitment rate* will be measured as the proportion of patients eligible to participate who are randomized to the trial; successful accrual will be defined as ≥ 80%; we anticipate achieving enrolment of 100 patients within 50 weeks with an average of 2 patients enrolled per week. Screening logs will be reviewed on a weekly basis to determine eligibility rates.
3. *Protocol adherence* will be measured as the proportion of instances when ChloraLock^TM^ is used during flushing and locking procedures; since this will be difficult to capture in a busy ICU setting, flushing and locking logs will be used as a surrogate to capture adherence. Successful protocol adherence will be ≥ 90% compliance with device use.
4. *Level of comfort with the trial protocol* staff comfort with ChloraLock^TM^ and the trial protocol will be measured using repeated surveys throughout the duration of the trial. Vetted suggestions will be incorporated into the protocol.

The ChloraLock^TM^ pilot trial also includes secondary, clinical outcomes related to the antimicrobial efficacy of the device and prevention of central line infections.

1. *Central line colonization*: Rates of central line colonization in each study arm will be documented. We will define central line colonization as a positive central line culture with concurrent negative peripheral stab culture. We will not order peripheral stab cultures to avoid harm, but will collect data from those ordered by the treating team.
2. *Bacteremia*: Rates of bacteremia in each study arm will be documented. We will define bacteremia as a positive peripheral stab culture and correlations will be done with central line cultures.
3. *Clinical end points* will be documented for each study arm:
  - LOS in ICU
  - LOS in hospital
  - ICU mortality at 28 days
  - Hospital mortality at 28 days

### Survey development

To determine the comfort level of ICU nurses with the ChloraLock^TM^ device and trial protocol, we will administer a paper-based survey to nurses who treat a study participant (see Additional file 5). We developed a nine-item survey to assess the ease of study-related tasks, time for device use, and effectiveness of educational activities and support materials. These domains were selected after consultation with the ICU educator and ICU nurse champions for the ChloraLock^TM^ study. The survey was pilot tested using a convenience sample of six ICU RNs, and further refinement of the survey involved clinical sensibility testing using a convenience sample of six ICU RNs. Feedback was provided on appropriateness, redundancy, and completion time of the tool.

### Participant timeline

**Fig. 2:**
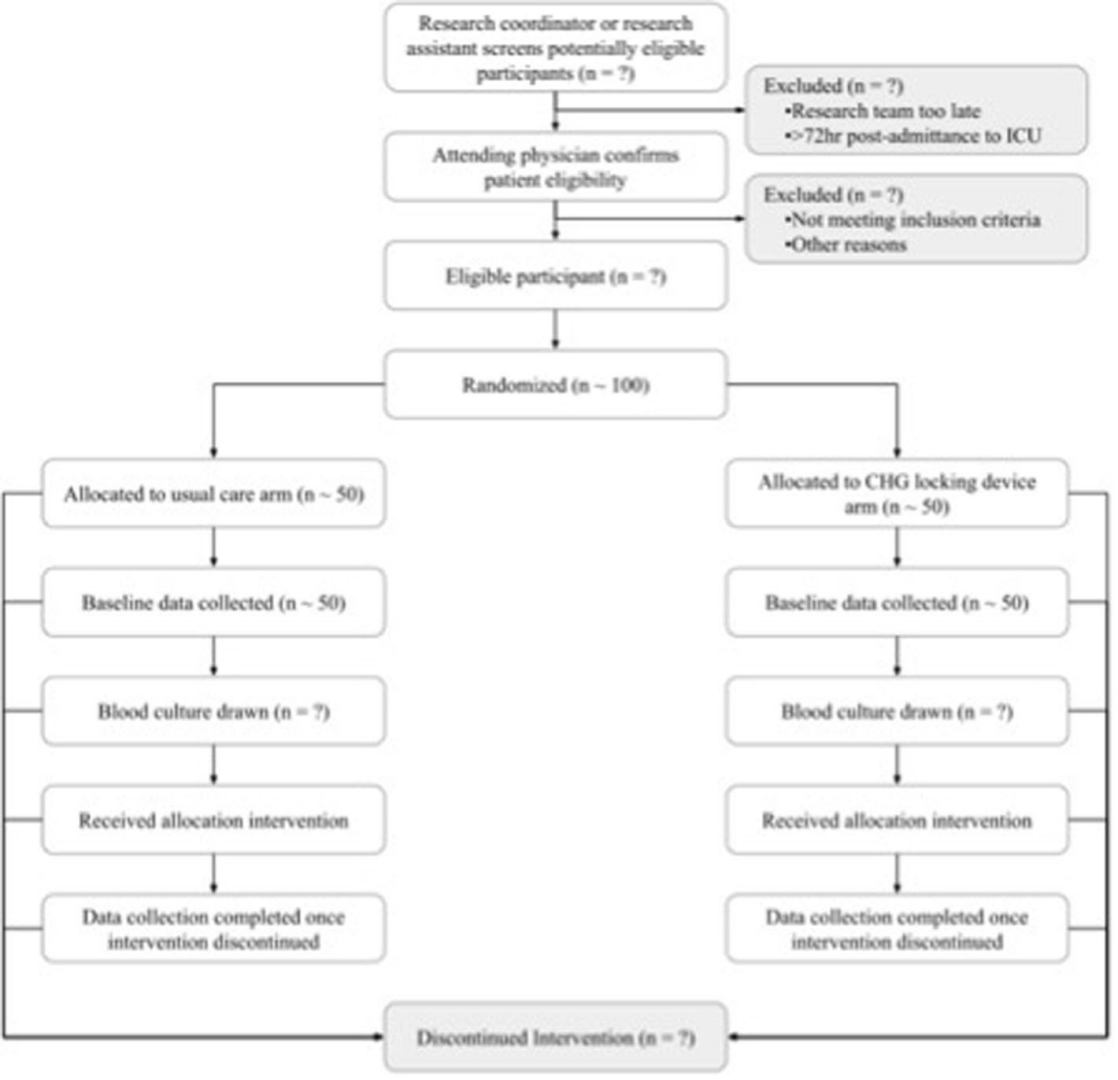
Flow of participants

Participants will be enrolled in the trial from the time of randomization until they leave the ICU. As shown in Fig. 2, data will be collected for the duration of participant ICU admission. For all participants, blood cultures will be drawn at baseline and every 48 h until all CVCs have been discontinued. For patients allocated to CHG locking device, device use will be discontinued when the patient leaves the ICU or all venous catheters have been discontinued.

### Sample size

The target sample size for the ChloraLock^TM^ pilot trial is 100 participants, with 50 participants randomized to each group. It is not possible to know the standardized effect size of our proposed intervention. Our best guess is to refer to other trials; however, trials that utilize our device do not exist. To err on the side of caution, we made the conservative assumption of having a very small effect size. A pilot study of such effect size is recommended to enroll 50–75 in each treatment arm as per the non-central t-distribution approach. Our target sample size is consistent with current guidelines on sample size calculation for pilot RCTs [36, 37] and the available funding from the sponsor.

### Screening and recruitment

Patients in three ICUs in one hospital will be screened for eligibility daily excluding weekends by the research staff and eligibility confirmed by the treating team. Once a patient is deemed to satisfy all inclusion and none of the exclusion criteria, the research staff will approach either the patient or the SDM on file to provide detailed information about the study and seek consent for participation (Additional file 6). It will be made clear that study participation is voluntary and refusal to participate will not impact the medical care provided. If the patient is unable to communicate and the SDM is not present in the ward, consent may be obtained from the SDM via telephone if witnessed by another staff member. Once written informed consent has been obtained, the research team will unseal an opaque envelope in sequence to randomize patients to receive usual care or CHG locking device. Screening and enrolment will conclude once our target sample size is reached. A de-identified record of all patients who were deemed eligible for enrolment but were not randomized will be kept on file. The following are potential and anticipated reasons: (1) The patient or SDM declined consent; (2) The patient is unable to give consent or the SDM is not available; and (3) The ICU physician declined consent.

### Allocation and blinding

Randomization will be done in block sizes of 2, 4, 6, and 8 in a fixed 1:1 ratio by means of a statistical computer software, R v3.4.2. Using the list generated by this software, 100 opaque security envelopes containing a note indicating either ‘ChloraLock’ or ‘Standard Care’ will be prepared and sequentially numbered. Envelopes will be sealed by a third party member. No members of the study staff will be privy to the allocation sequence generated. Upon obtaining informed consent, the research staff will open the security envelope that corresponds with the patients study ID to determine allocation. It will not be possible to blind the research staff, physicians, and bedside nursing staff of participant assignment since these individuals need to be aware of allocation to implement the intervention. The principal investigator will be blinded to the study outcomes throughout the duration of the trial.

### Data collection and management

Data collection will be performed by a research assistant trained in the use of Data Collection Forms. Participant demographic data and ChloraLock^TM^ pilot trial data will be extracted from their electronic health records, which will be accessed via the electronic patient database at Hamilton Health Sciences. Data will first be recorded in paper-based Case Report Forms (CRFs) that will be stored in a locked office at the Thrombosis and Atherosclerosis Research Institute (TaARI). Data will then be entered in electronic CRFs in REDCap, with pre-programmed range checks to help ensure data quality. Paper-based screening logs will be kept in a locked office and outcome data will be stored on the Team Sepsis database, both located at TaARI. Routine source data verification will occur for all data collected including eligibility, daily data, protocol violations, adverse events (AEs), and serious adverse events (SAEs). Participant data will be retained for 25 years stored off-site using Iron Mountain.

### Statistical analysis

Simple descriptive statistics will be used for feasibility outcomes and baseline demographics (Table 3). Continuous data will be tested for normality using histograms, Normal Q-Q plots, skewness, kurtosis, and the Shapiro–Wilk test. Normally distributed data will be summarized using mean and standard deviation, non-normal data will be summarized using median and interquartile range, and categorical variables will be summarized using number and percent proportion. The two treatment arms will be compared by simple statistics as the study may not be powered for the secondary outcomes. However, we will test group differences with either the independent-samples t-test or the Mann–Whitney U test. Somers’ delta (*d*) will be used to identify and measure the strength and direction of association that exists between two ordinal variables, particularly for the number of positive cultures. If there is a large treatment effect between groups, this may aid the sponsor in proceeding with larger RCT as required for regulatory approval within other countries. All statistical tests will be conducted using SPSS version 24 (IBM Corp, Armonk, NY).

**Table 3:** Summary of primary and secondary outcomes for the ChloraLock^TM^ pilot trial

Pilot trial outcomes	Analysis	Pass threshold
Primary outcomes		
1. Consent rate: patients and SDMs approached who consent to randomization	Proportion	≥ 80%
2. Recruitment rate: eligible patients who are randomized	Proportion	≥ 80%
3. Protocol adherence: times ChloraLock^TM^ is used in conjunction with usual locking procedures	Proportion	≥ 90%
4. Comfort: staff comfort level with the device and trial protocol	Descriptive	NA
Secondary outcomes		
1. Central line colonization: assessed for each arm as positive central line culture and negative peripheral poke	Proportion	NA
2. Bacteremia: assessed for each arm as positive stab cultures	Proportion	NA
3. Clinical end points: LOS in ICU, LOS in hospital, ICU mortality at 28 days, hospital mortality at 28 days	Median or mean proportions	NA

Given the small sample size and the focus on feasibility outcomes, there will be no subgroup analyses or interim analyses. We will adhere to the intention to treat principle.

### Data monitoring committee

Data monitoring committees (DMCs) are often established to independently monitor the safety and treatment efficacy of a clinical trial. However, the decision was made not to establish a DMC to oversee the ChloraLock^TM^ pilot trial due to the minimal risk associated with participation.

### Interim analyses and auditing

There will be no interim analyses of study data during the trial to avoid biases and over-interpretation; all analyses will be carried out once the trial has been completed. There are no scheduled audits of the ChloraLock^TM^ pilot trial; however, the trial may be subject to audit by the REB or Health Canada.

### Harms

All SAEs will be reported to the Hamilton Integrated Research Ethics Board (HiREB). If a clinician caring for a study participant believes an AE is trial related this will be documented. For the first 15 trial participants all AEs will be assessed for unexpected events. Data regarding all AEs will be collected and reported. Any SAE (anaphylaxis, sudden death with use of device) will be reported to Health Canada and the sponsor.

### Confidentiality and data management team

Only those participant identifiers determined to be necessary will be collected and participants will be assigned a unique code number for trial purposes. Participant identifiers will be kept separate from all other trial data collected. The file linking code numbers with participant identifiers will be password protected and stored in the Team Sepsis Database. The investigators, research coordinator, and research assistant will have access to the trial dataset.

### Communication of trial results

The plan for communication of trial results includes presentations at national and international scientific conferences, and publication in a peer-reviewed journal. Authorship on any manuscript disseminating study results will be determined in accordance with the standard operating procedures and guidelines set out by the Canadian Critical Care Trials Group. There will be no medical or professional writers involved in manuscript preparation.

## Discussion

Various interventions have been investigated to prevent central line infections in ICU patients [24–27]. Previously studied interventions have had limited success with potentially harmful complications such as increased thrombotic events and antibiotic resistance; thus, no interventions have been broadly adopted for clinical use [28, 38]. We describe a pilot RCT evaluating the feasibility of ChloraLock^TM^, a new medical device that diffuses CHG into the locking solutions of CVCs with the aim of preventing central line infection in ICU patients. In vitro and preclinical animal trials have validated the antimicrobial efficacy of ChloraLock^TM^ [33], but the proposed study will be the first to assess the feasibility and efficacy of the device in a clinical population. This study will generate preliminary data on the efficacy of a CHG locking device for the prevention of central line infection and inform the feasibility of conducting a large-scale RCT in terms of optimal strategies for patient recruitment, consenting procedures, and protocol adherence.

Device trials face unique barriers to protocol uptake and adherence, and high compliance rates are difficult to achieve for complex interventions in ICU settings [39, 40]. Use of ChloraLock^TM^ requires nurses to perform additional steps during routine intravenous line care, and it is unknown how nurses involved in the trial will operationalize the protocol for optimal compliance. The challenges associated with device trials support the need for an initial pilot trial that will be used to refine the ChloraLock^TM^ protocol before proceeding to a larger RCT. Findings regarding weekly and monthly recruitment yields will be used to develop a recruitment strategy to feasibly enrol an adequately powered definitive RCT. The findings from this pilot trial will also allow us to identify the most effective strategies for ensuring high levels of protocol adherence. Furthermore, we anticipate that feedback from bedside nursing staff will help identify any major barriers to the device use and protocol adherence.

Medical device trials are notorious for their lack of strict regulations and the conduct of well-designed RCTs for medical devices is sparse [41]. This protocol describes a scientifically rigorous RCT that attempts to address the need for well-designed medical device trials and generate high-quality evidence on benefits and harms of a proposed medical device.

## Trial status

The authors are still actively recruiting participants at the time of manuscript submission.

## Supplementary information

**Additional file 1.** Populated SPIRIT checklist.

**Additional file 2.** Items from the World Health Organization Trial Registration Data Set.

**Additional file 5.** Survey of ICU nurses’ perceptions of the ChloraLock^TM^ device and protocol.

**Additional file 6.** REB approved Consent Form.

## Data Availability

Data and materials will be made available upon written request.

## Abbreviations

AE: Adverse event
CFU: Colony-forming units
CHG: Chlorhexidine gluconate
CLABSI: Central line-associated bloodstream infection
CRF: Case report form
CVC: Central venous catheter
DMC: Data monitoring committee
HAI: Hospital-acquired infection
HGH: Hamilton General Hospital
HiREB: Hamilton Integrated Research Ethics Board
ICU: Intensive care unit
LOS: Length of stay
NS: Normal saline
PI: Principal investigator
REB: Research Ethics Board
SDM: Substitute decision maker
SPIRIT: Standard Protocol Items: Recommendations for International Trials
TaARI: Thrombosis and Atherosclerosis Research Institute
