# Supplementary material for: Chlorhexidine locking device for central line infection prevention in ICU patients: protocol for an open-label pilot and feasibility randomized controlled trial
Source: Pilot Feasibility Stud. 2020 Feb 18;6:26. doi: 10.1186/s40814-020-0564-9 (PMC7027059; doi:10.1186/s40814-020-0564-9)
Supplement: Supplementary file 4 — Additional file 2. Items from the World Health Organization Trial Registration Data Set. [file 40814_2020_564_MOESM2_ESM.pdf]

Multimedia Appendix 1: Items from the World Health Organization Trial Registration Data Set

**Data Category**	**Information**
Trial Identifier	ClinicalTrials.gov NCT03309137
Date of registration	October 13, 2017
Secondary Identifying Numbers	HIREB Project Number: 3654
Sources of Monetary and Material Support	ICU Medical<br>ATTWILL
Primary Sponsor	ICU Medical
Public Title	Pilot trial for a chlorhexidine locking device
Scientific Title	Chlorhexidine locking device for central line infection prevention in ICU patients: Study protocol for an open-label, randomized, pilot feasibility trial
Countries of Recruitment	Canada
Health Condition Studied	Central-Line Associated Bloodstream Infection (CLABSI)
Intervention	Intervention group: CHG locking device, ChloraLock^{TM}
	Control group: usual care
Inclusion and Exclusion Criteria	Inclusion: Adults (>18) within 72 hours of ICU admission and with a central line expected to remain in situ for at least 72 hours.
	Exclusion: Suspected infection with antibiotic treatment, chronic indwelling catheters, poor prognosis, and chlorhexidine allergy.
Study Type	Interventional, randomized, open-label, feasibility study.
Date of First Enrollment	November 2017
Target Enrollment	100
Recruitment Status	Recruiting
Primary Outcomes	Recruitment rate, consent rate, protocol adherence, comfort level.
Secondary Outcomes	Central line colonization, bacteremia, clinical end points.
