# Supplementary material for: Chlorhexidine locking device for central line infection prevention in ICU patients: protocol for an open-label pilot and feasibility randomized controlled trial
Source: Pilot Feasibility Stud. 2020 Feb 18;6:26. doi: 10.1186/s40814-020-0564-9 (PMC7027059; doi:10.1186/s40814-020-0564-9)
Supplement: Supplementary file 5 — Additional file 5. Survey of ICU nurses’ perceptions of the ChloraLockTM device and protocol. [file 40814_2020_564_MOESM5_ESM.pdf]

CHLORALOCK STUDY -NURSES SURVEY

You are being invited to participate in a brief survey about the Chloralock device. We are seeking to gather information about your **first-hand experiences** with the Chloralock study.

VOLUNTARY PARTICIPATION

- You are not obligated to complete this survey.
- You may skip questions you are not comfortable answering.
- Consent to participate in this survey is given by your return of a completed survey.
- All of your responses will remain **confidential**.

- Upon completion of the survey please enter your name into a draw for a $15 coffee card by sending an email to Yasmin Sivji at Yasmin.Sivji@taari.ca, or Makena Pook at pookm@mcmaster.ca.
- Printed copies of completed surveys can be placed inside the envelope in the black wire basket on the wall behind where the charge nurse sits.

1. **Did your patient receive?** (check one response)

◯ Standard care (no device)
◯ Chloralock device (specify the number of patients you treated with the device)
____

2. **Please rate how difficult or easy you found the following tasks.** (check one response for each variable)

	Very Difficult	Difficult	Neither Difficult nor Easy	Easy	Very Easy	NA (standard care)
Using Chloralock device	◯	◯	◯	◯	◯	◯
Getting help from the study staff	◯	◯	◯	◯	◯	◯

3. **To what extent do you agree or disagree with the following statement, "I feel comfortable using the Chloralock device"?** (check one response)

Strongly Disagree	Disagree	Neither Disagree nor Agree	Agree	Strongly Agree	NA Standard Care
◯	◯	◯	◯	◯	◯

4. **Please estimate how much additional time was required for the following tasks:** (check one response for each variable)

	<1 minute	1-5 minutes	>5 minutes	NA (standard care)
Each use of the Chloralock device	○	○	○	○
Each documentation in utilization log	○	○	○	○

5. **How did using the Chloralock device and documentation impact your workload?** (check one response)

Significantly Increased	Increased	Neither Increased nor Decreased	Decreased	Significantly Decreased
○	○	○	○	○

6. **If you were ever unable to use the Chloralock device, please indicate the reason(s) why.** (check all that apply)

- ☐ Urgent access to IV required
- ☐ Unaware patient enrolled in the study
- ☐ Not yet in-serviced on study protocol
- ☐ Uncomfortable with protocol
- ☐ Other
  ____
- ☐ Device not available
- ☐ Missing green label/sticker
- ☐ Time constraints
- ☐ Forgot
- ☐ NA (always used device)

7. **Do you have any concerns about study coordinators taking blood cultures?**

- ○ Yes
- ○ No

8. **If your patient was receiving dialysis did this impact your ability to follow study protocol?**

- ○ Yes
- ○ No
- ○ NA (did not have a dialysis patient)

9. **Please indicate how effective you found each of the following resources for instructional and supportive purposes:** (please check one response for each variable)

	Very Ineffective	Ineffective	Neither Ineffective nor Effective	Effective	Very Effective
Initial In-servicing	◯	◯	◯	◯	◯
Posters and diagrams	◯	◯	◯	◯	◯
Chart with flushing and locking volumes	◯	◯	◯	◯	◯
Colour coding system (use of green stickers)	◯	◯	◯	◯	◯
Utilization log (green sheet)	◯	◯	◯	◯	◯
Availability of research support staff	◯	◯	◯	◯	◯
Ease of identifying patients in the study (i.e. Kardex and med profile stickers)	◯	◯	◯	◯	◯
Availability of study materials (i.e. Chloralock device)	◯	◯	◯	◯	◯

Please provide feedback on the following (OPTIONAL):

10. What did you like about the Chloralock device?

____

11. Do you have concerns regarding the Chloralock device?

____

12. Do you have any suggestions for design improvements of the Chloralock device?

____

13. Do you have any suggestions for improvements of the implementation of the study protocol?

____

Demographics (OPTIONAL):

Please tell us a little bit about yourself. Your information will be kept entirely confidential.

14. **Sex:**

◯ Female
◯ Male
◯ Other

15. **What is your current Employment status?**

◯ Full time
◯ Part time
◯ Casual

16. **How many years of nursing experience do you have (include current year)?**

◯ 1-5 years
◯ 6-10 years
◯ 11-15 years
◯ >15 years

17. **How many years of nursing experience do you have in the ICU unit (include current year)?**

◯ 1-5 years
◯ 6-10 years
◯ 11-15 years
◯ >15 years

Thank you for participating in the survey!

Makena Pook, Nasim Zamir, Ellen McDonald and Alison Fox-Robichaud.
