# Supplementary material for: Chlorhexidine locking device for central line infection prevention in ICU patients: protocol for an open-label pilot and feasibility randomized controlled trial
Source: Pilot Feasibility Stud. 2020 Feb 18;6:26. doi: 10.1186/s40814-020-0564-9 (PMC7027059; doi:10.1186/s40814-020-0564-9)
Supplement: Supplementary file 6 — Additional file 6. REB approved Consent Form. [file 40814_2020_564_MOESM6_ESM.pdf]

CHG-Lock™ Antiseptic Device for Bacteremia Prevention in CV-ICU Patients: An Open Label Randomized Feasibility Study

LEGALLY AUTHORIZED REPRESENTATIVE INFORMATION AND CONSENT FORM

Principal Investigator: Dr. Alison Fox-Robichaud, MD, MSc, FRCPC Office: (905) 521-2100 x 40742
Funding agency: ICU Medical

BACKGROUND

Your family member is being invited to participate in a research study. This document describes the research study. Your family member could take part in this study, if you agree.

Patients who are in the Intensive Care Unit (ICU) have intravenous (IV) lines. IV lines are needed for medication and fluid administration as well as vital sign monitoring and taking blood specimens for laboratory analysis.

If your doctor suspects your loved one will be in the ICU for longer than a few days and s/he has IV lines, s/he may be eligible for the study.

Bacteria in the blood are also known as bacteremia. Bacteremia can over power the body and cause problems such as organ failure i.e. kidney failure - need for dialysis; respiratory failure - need for the breathing machine, and the need for medication to bring the blood pressure up to normal. In severe, situations bacteremia can lead to death. The rate of bacteremia in ICU or hospitalized patients is 5-10%.

Once you understand the study, you will be asked to sign this form if you wish your loved one to participate. Your decision to participate is voluntary. Your family member's care will not be adversely affected in any way if you choose not to participate. If you have questions about the study, at any time, please call Dr. Alison Fox-Robichaud 905 521-2100 ext 40742. You may talk to any of your doctors about this study.

PURPOSE

When not in use, IV lines are said to be "locked". A normal saline solution is added to the catheter of the IV at regular intervals and sealed off with a sterile cap. The purpose of this study is to determine if there is less bacteria in the blood (bacteremia) when using an antiseptic device with IV lines that are "locked" as compared to IV lines that are locked without the antiseptic device, (usual care). In addition to looking for bacteria in the blood, we want to know if we can enroll patients in the study and the device can be used consistently for those patients who are getting the device. This study may help doctors and nurses prevent infections in the blood and the negative effects associated with bacteremia. such as organ failure and possibly death.

STUDY GROUP ALLOCATION (RANDOMIZATION)

Your family member will be put into one of two groups through a process called Randomization. The decision about what group s/he will be in will be determined by a computer generated process. This randomization process takes the bias out of the hands of the research team. The 2 groups are a) patients who will receive the antiseptic device (CHG-Lock™) and b) patients who will receive routine care with all IVs. 100 patients will be in the study. Half of the study participants (50) will receive the antiseptic device and the other half (50) will have routine or usual care of the IV lines. At the end of the study we will compare both groups to see who had fewer rates of bacteria in the blood.

The study will be conducted in the Hamilton General Hospital.

PATIENT RESPONSIBILITIES

When an Intravenous (IV) line is not in use we will use a normal saline flush at regular intervals to keep it patent (open). This process is known as locking the IV. If your family member is in the antiseptic device group, s/he will have a new antiseptic device each time the IV is locked for every IV line. If s/he is in the

routine care arm, the IVs will be managed as part of the usual care for IVs at Hamilton General Hospital. The study protocol will continue for as long as your family member remains in the Intensive Care Unit. When s/he leaves the ICU the study protocol is complete. S/he will not have to come back to the hospital for a follow up visit.
Some preliminary information pertaining to your loved one's medical history will be obtained. We will obtain blood cultures to determine if there are bacteria in the blood, at the beginning of the study and every 2 days (48hrs). If the medical teams suspects bacteria in the blood or an infection they will obtain additional blood cultures as needed and prescribe the treatment that is needed.
We will obtain blood cultures through an existing arterial line or large IV line. These lines are used for fluid and medication administration, monitoring and obtaining blood work without causing any discomfort. The amount of blood we will need is 1.4 tablespoons at the beginning of the study and every 2 days as long as your family member is in the ICU. If your family member has a large IV, called a Central Line, and it is removed as part of routine care, we may sent the catheter to the lab to determine if there are bacteria in the catheter. When bacteria are present in the blood or the IV catheter the medical team will treat your family member with the treatment that is needed.

BENEFITS, RISKS, DISCOMFORTS
Participation is voluntary and should you or your family member choose not to participate, there will be no negative impact on the care you receive. As mentioned the blood specimens will be taken through an existing line and will not cause discomfort. Once the blood has been drawn, it will be sent to the laboratory and the ICU medical team will have access to the results.

WHAT ARE THE POSSIBLE BENEFITS FOR ME AND/OR FOR SOCIETY?
As the Canadian population ages, the number of people who will experience an ICU stay will increase dramatically. Your participation may help us discover in the future, ways to help prevent bacteremia in ICU patients and the negative consequences associated with blood infections.

CONFIDENTIALITY
Your family member's personal health information will not be shared with anyone except with your consent or as required by law. All personal information such as name, address, phone number, and family physician's name will be removed from the data and will be replaced with a number. A list linking the number with your loved one's name will be kept separate in a secure place. The data, with identifying information removed, will be securely stored in a locked office in the research office. The study data will be retained for 25 years.

For the purposes of ensuring the proper monitoring of the research study, it is possible that a member of the hospital Research Ethics Board, a Health Canada representative, or a study monitor may look at your family member's research data and medical records. However, no records which identify him/her by name or initials will be allowed to leave the Hamilton General Hospital or McMaster University. By signing this consent, you authorize such access.

When the results of the study are published, your loved one's name will not be used and no information that discloses his/her identity will be released or published without your specific consent to the disclosure. However, it is important to note that this original signed consent form and the data that follows may be included in your family member's health record.

IF I DO NOT WISH TO TAKE PART IN THE STUDY, ARE THERE OTHER CHOICES?
Your decision for your loved one to take part in this study is strictly voluntary. You are free to refuse or to change your mind (withdraw your consent) at any time. If you choose to withdraw him/her from the study, you will have the option of allowing continued collection of clinical and outcome data, or requesting that no further data be collected aside from survival data. The decision to withdraw from the study or not to take part in the study will in no way compromise the care that your loved one will receive. Simply let your loved one's nurse or doctor that you no longer want your family member to be in the study and they will contact the research team to inform them. You may contact the research team yourself. The names and phone numbers of the research team are at the end of this form.

COMPENSATION
You or your family member will not be paid for participation in this study. Study participation will not involve any additional costs to you or your health care insurer (the Ontario provincial government).

WRITTEN CONSENT
I have read and understand the information about this study. I have been given the opportunity to ask questions and will receive a copy of the signed consent form. I can sign the consent form in person or via FAX.

PATIENT or LEGALLY AUTHORIZED REPRESENTATIVE

Name of Patient (Please Print)	Signature	Date	__:__ Time

If patient is incapable, relationship of legally authorized representative to participant: ____________________

Name of Patient (Please Print)	Signature	Date	__:__ Time

RESEARCH PERSONNEL

I have explained the study to the above patient or Next of Kin, the purpose, the potential benefits, and possible risks associated with participating in this study. I have answered all questions that have been raised.

Name of Person Obtaining Consent (Please Print)	Signature	Date	__:__ Time

WITNESS Only when phone consent or when translation is required.

I was present when the information in this form was discussed with the participant/substitute decision maker. I believe the participant/substitute decision maker understands what is involved in this study

Name of Witness (Please Print)	Signature	Date	__:__ Time

PRINCIPAL INVESTIGATOR

Name of Principal Investigator (Please Print)	Signature	Date	__:__ Time

This study has been reviewed by the Hamilton Integrated Research Ethics Board (HIREB). The HIREB is responsible for ensuring that participants are informed of the risks associated with the research, and that participants are free to decide if participation is right for them. If you have any questions about your rights as a research participant, please call the Office of the Chair, Hamilton Integrated Research Ethics Board at 905.521.2100 x 42013.

If I have any further questions about my ongoing participation in the study, I may contact:

Dr. Alison Fox-Robichaud, MD
Principal Investigator
(905) 521-2100 ext 40742

Ellen McDonald RN
Research Coordinator
(905) 521-2100 ext 40787
